# A Deep Learning Approach for the Photoacoustic Tomography Recovery From Undersampled Measurements

**DOI:** 10.3389/fnins.2021.598693

**Published:** 2021-02-24

**Authors:** Husnain Shahid, Adnan Khalid, Xin Liu, Muhammad Irfan, Dean Ta

**Affiliations:** ^1^Department of Electronic Engineering, Fudan University, Shanghai, China; ^2^Department of Software Engineering, Northeastern University, Shenyang, China; ^3^Academy for Engineering and Technology, Fudan University, Shanghai, China

**Keywords:** photoacoustic tomography, deep learning, compressed sensing, under-sampled measurements, image reconstruction

## Abstract

Photoacoustic tomography (PAT) is a propitious imaging modality, which is helpful for biomedical study. However, fast PAT imaging and denoising is an exigent task in medical research. To address the problem, recently, methods based on compressed sensing (CS) have been proposed, which accede the low computational cost and high resolution for implementing PAT. Nevertheless, the imaging results of the sparsity-based methods strictly rely on sparsity and incoherence conditions. Furthermore, it is onerous to ensure that the experimentally acquired photoacoustic data meets CS’s prerequisite conditions. In this work, a deep learning–based PAT (Deep-PAT)method is instigated to overcome these limitations. By using a neural network, Deep-PAT is not only able to reconstruct PAT from a fewer number of measurements without considering the prerequisite conditions of CS, but also can eliminate undersampled artifacts effectively. The experimental results demonstrate that Deep-PAT is proficient at recovering high-quality photoacoustic images using just 5% of the original measurement data. Besides this, compared with the sparsity-based method, it can be seen through statistical analysis that the quality is significantly improved by 30% (approximately), having average SSIM = 0.974 and PSNR = 29.88 dB with standard deviation ±0.007 and ±0.089, respectively, by the proposed Deep-PAT method. Also, a comparsion of multiple neural networks provides insights into choosing the best one for further study and practical implementation.

## Introduction

Photoacoustic tomography (PAT) is a coupled-physics imaging modality that allows noninvasive, quantitative, and 3-D imaging of biological and biochemical processes in living small animals. However, fast PAT imaging remains an open problem for the research community. Until now, multiple compressed sensing (CS)–based methods have been proposed, and they contribute to recovering the original signals in a few measurements but with highly iterative and computational cost (Foucart and Rauhut, 2013). Inspired by CS theory, Provost and Lesage (2009) applied CS to PAT for small animal imaging by the highly computational iterative CS methods. Moreover, in the context of sparsity, some work has also been accomplished on data-dependent dictionaries (Mallat, 1999; Aharon et al., 2006; Duarte-Carvajalino and Sapiro, 2009) to solve the PAT imaging problem, but these techniques wane the recovery performance. In Guo et al. (2010), employed a CS modality to implement *in vivo* PAT imaging. However, it must be noted that, to obtain the optimal imaging results, the sparsity-based methods are strictly relying on sparsity and incoherence conditions (Provost and Lesage, 2009). Furthermore, it is arduous to ensure that the experimentally acquired photoacoustic data comply with the prior requirements of CS. In other words, when encountering complex experimental conditions, the acquired photoacoustic data may not be precisely sparse in a fixed basis (transform). Generally, the smooth images are sparse on a Fourier basis. In contrast, the piecewise-smooth images and the images with discontinuities along the edges are sparse on wavelet and curvelets bases, respectively (Candes and Donoho, 2004; Provost and Lesage, 2009). As a result, it is a challenging task to find the exact basis to make the photoacoustic data sparse. To some extent, it limits the application of the sparsity-based method for *in vivo* experiments.

Recently, deep learning is dominating by significantly facilitating the performance of multiple tasks, including classification (Wang et al., 2020), segmentation (Ronneberger et al., 2015), and reconstruction, etc. (Zhang and Dong, 2020). In medical imaging fields, e.g., magnetic resonance imaging (MRI) and computed tomography (CT) etc., convolutional neural networks (CNN) have been used to improve the imaging quality (Han et al., 2016; Hawn et al., 2016; Wang et al., 2016b; Chen et al., 2017) further. Additionally, Dreier et al. (2017) applied the learning-based method to solve the PAT’s limited-view problem (extends the limited views). In Antholzer et al. (2018), share the sparse data problem’s views, implement PAT imaging by using filtered back projection (FBP), and diminish the artifacts by U-Net. The similar artifacts problem has also been resolved by Davoudi et al. (2019) with the same U-Net network on PAT. In CS-alone techniques, these methods are highly iterative and computationally expensive (Provost and Lesage, 2009). On the other hand, utilizing only the deep learning algorithm demands the structured data as an input (e.g., sparse). Especially in the case of usage of high-resolution data for training, the image sizes are larger and so is the network complexity. Hence, to avoid such a situation, the data needs to be converted into small slices or sparse domains (to avoid slicing) and use the multiple fully connected layers to recover the image (Lliadis et al., 2018). Apart from this, these approaches require the network to be trained and change the parameters according to the sampling ratio every time as they usually use the defined measurement matrix given in Eq. (7). Besides this, random sampling can provide better quality, but it can only be applied if the image is in the sparse domain (Provost and Lesage, 2009). Hence, combining the inverse CS and deep learning helps to get rid of iterative computational methods, diminish the prerequisite of CS, and improve image quality. To our knowledge, this is the first paper using an amalgamation of inverse CS and deep learning (Deep-PAT) for photoacoustic data and focusing on recovering high-resolution PAT imaging in a few measurements even if the experimental data does not follow the prerequisite conditions of CS (sparsity and incoherence). To address the above problems, the combined method is utilized, which diminishes the above limitations for recovery.

The paper is organized as follows. In Section 2, the reconstruction methods are presented, including the PAT imaging model, the sparsity-based techniques, the proposed Deep-PAT method using multiple neural networks, and details about quantitative analysis. Section 3 explains the experimental materials, data preprocessing, and network training. The details about the results, discussions, and comparison are presented in Section 4. Finally, we conclude with a summary in Section 5.

## Reconstruction Methods

### PAT Imaging Model

Photoacoustic tomography allows implementing high-resolution imaging *in vivo* by combining optical absorption contrast and high ultrasound resolution. In comparison with classical imaging modalities, PAT can achieve higher spatial resolution at depth. According to Liu et al. (2012), the imaging model of PAT can be formulated by a heterogeneous wave equation as follows:

(1) $$\nabla^{2\,p\,\left(r,t\right)}-\frac{1}{c^{2}}\,\frac{\partial^{2\,p\,\left(r,t\right)}}{\partial t^{2}}=-\frac{\beta }{C}\,\frac{\partial H\,\left(r,t\right)}{\partial t},$$

Where *c* is sound velocity, *p*represents pressure, t is the time, β provides information isobaric volume expansion coefficient, and *C* is related to heat capacity. The right-hand side of Eq. (1) depends on the heat source *H*(*r*,*t*) that can be written as the product of the absorbed optical energy density *A*(*r*) and a temporal function of illumination *I*(*t*) (Wang et al., 2016a),

(2) H⁢(r,t)=A⁢(r)⁢I⁢(t).

For PAT imaging, the main concern is to recover *A*(*r*) from the pressure measurement *p*(*r*,*t*).

### Compressed Sensing Methods

According to CS theory, the data consisting of *N* samples can be transformed into a sparse domain by finding a suitable sparse transform ψ, as follows:

(3) θ=ψ⁢x,

Where θ belongs to the transformed sparse image and *x* is the original image. If *x* contains *N* pixels, then sparsity is defined as ||*θ*||*l*_0_ ≪ *N* and ℓ_0_ norm is simply the nonzero coefficients. The main objective of CS is to recover the image *x* through measurement data from the imaging system. Assuming that the measurement data *y* is obtained through a measurement matrix *K*, we have the following relation:

(4) y=K⁢x,

In terms of CS, the photoacoustic data can be reconstructed by solving the following convex optimization problem (Wang et al., 2016a):

(5) $$\text{min}\,{||\theta ||}_{l_{0}}\,s.t\;y=K\,\psi^{-1}\,\theta ,$$

Where ψ donates the suitable sparse transform and _*K*_ is related to the physical imaging system. To use CS effectively, kψ must be a CS matrix (Provost and Lesage, 2009). Note that minimizing ℓ_0_ is a combinatorial problem and not applicable if one wants to recover the high-resolution images. To overcome these limitations, it can be mathematically seen that theℓ_1_ minimization problem is equivalent for most *Kψ* if the solution is sufficiently sparse (Donoho, 2006). Therefore, the mathematical model in Eq. (5) is derived as

(6) $$\text{min}\,{||\theta ||}_{l_{l}}\,s.t\,y=K\,\psi^{-1}\,\theta ,$$

To implement CS reconstruction for PAT, the derivation of the measurement matrix is critical. Based on Eq. (1) and back-propagation theory (Meng et al., 2012), the measurement matrix is directly derived as follows (Donoho, 2006):

(7) $$K\,{\left(h,t\right)}_{\left(i,j\right)}=\frac{1}{2\,\pi \,c}\,\delta \,\left(t-\frac{\left|r_{i,j}-r_{h}\right|}{c}\right),$$

where *h* = 1,2……,*p*, *t* = *s*Δ*t*, and *s* = 1,2,…….,*q*_*s*_. According to Donoho (2006), the above measurement matrix in the frequency domain could be written as follows:

(8) $$K\,{\left(h,n\right)}_{\left(i,j\right)}=i\,c\,k_{n}\,\frac{\exp\left(-i\,k_{n}\,\left|r_{i,j}-r_{h}\right|\right)}{\left|r_{i,j-r_{h}}\right|},$$

where *h* = 1,2……,*p*, and *n* = 1,2,…..*q*_*n*_. *r*_*i*,*j*_ represent the cartesian coordinates, *r*_*h*_ donates the transducer’s position, *p* considers the number of transducers, and *q*_*s*_ and *q*_*n*_ represent the sampling points in time and frequency domain, respectively.

As mentioned above, when facing complex experimental conditions, the acquired photoacoustic data may not be exactly sparse on a fixed basis, which is the initial prerequisite of the CS technique in context to get the exact reconstruction of PAT (Provost and Lesage, 2009). Hence, there is a constraint in utilizing CS, which needs to be diminished to acquire the data in just a few measurements. Even after finding the sparse basis ψ, there is another limitation that has been discussed earlier. That is, the matrix *Kψ* must be a CS matrix, which means that the matrix obtained from the product of the measurement matrix and the sparse basis must show a certain quantity of linear independency among a small group of columns or must fulfill the restricted isometric property (RIP) to retrieve the data efficiently.

According to the research (Cand‘es, 2008), (RIP) states that

(9) $$\left(1-\delta_{s}\right)\,{||\theta ||}_{2}^{2}\leq {||A\,\theta ||}_{2}^{2}\leq \left(1+\delta_{s}\right)\,{||\theta ||}_{2}^{2},0<\delta_{s}<1$$

For sparse vector θ, *δ*_*s*_ is a restricted isometric constant, and *A* is related to *Kψ*. Suppose we have an arbitrary sparse vector based on CS theory. To recover the vectors from measurements taken as *v* = *Ax*, one needs to ensure that it is possible to distinguish between measurements *v*_1_ = *Ax* and *v*_2_ = *Ax* of any two such vectors. If they are the same, it is not possible to distinguish and reconstruct them. Hence, for reconstructing the sparse vector efficiently from measurements taken with *A*, the restricted isometric property quantifies how well *A* contributes to performing that task. As our concern is real data, which is usually not sparse on a fixed basis (Kashyap, 2019), it cannot fulfill the RIP property and can lead to inefficacious reconstruction.

### Deep Learning Methods

**FIGURE 1:**
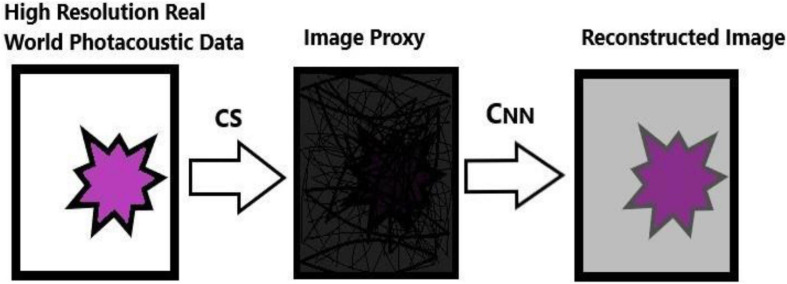
Summary of the proposed Deep-PAT Method.

**FIGURE 2:**
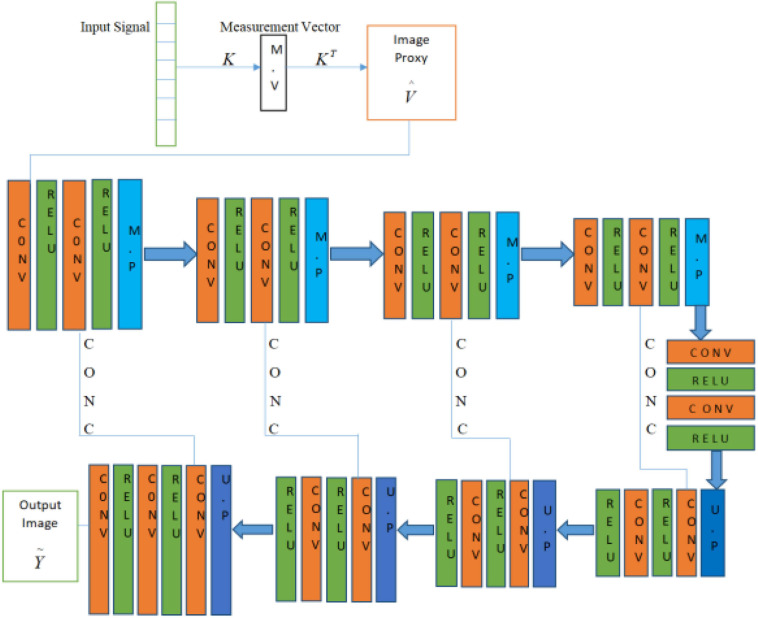
Deep-PAT based on simple U-Net for image reconstruction.

A deep learning method is proposed to overcome the limitation of the sparsity-based methods, which can recover the PAT imaging from undersampled data without making them sparse The summary of Deep-PAT is shown in Figure 1. Briefly, as an illustration in Figure 2, a compressed sensing approach is applied to the input data in *ℝ*^*N*×*N*^, which further converts the image data into measurement vector *v* (*ℝ*^*M*^) by multiplying with the random measurement matrix *K* with *M*≪*N*. After getting the measurement vector *v*, the fully connected layer is used to generate an image proxy $\hat{V}$. Note that, in this case, the output image may consist of the artifacts and the fuzzy object while the sparsity and RIP property conditions are being ignored. Eventually, the deep learning–based network is applied to remove the undersampled artifacts and recover the image object with high resolution.

In detail, the task of high-resolution PAT reconstruction can be described as a supervised machine learning problem. This context’s primary concern is to evaluate the mapping function *ξ*:*ℝ*^*M*×*M*^→*ℝ*^*N*×*N*^, which maps the input measurement vector *v* ∈ *ℝ*^*M*^ to the image proxy with artifacts and fuzzy object in the $\hat{V}$ ∈ *ℝ*^*N*×*N*^ space, which needs further processing to get the artifact-free and visible output in the $\tilde{Y}$ ∈ *ℝ*^*N*×*N*^ space. To design such a mapping function, one assumes that the artifact images (image proxy) *V*_*n*_ and artifact-free images *Y*_*n*_ combine to make a training data $T={\left(V_{n},Y_{n}\right)}_{n=1}^{N}$ pair.

Based on the neural network theory, the mapping function ξ is formulated as the training error

(10) $$E\,\left(T;\xi \right)=\sum_{n=1}^{N}e\,\left[\xi \,\left(V_{n}\right),Y_{n}\right],$$

Which is minimized as min[*E*(*T*;*ξ*)], where *e*:*ℝ*^*N*×*N*^×*ℝ*^*N*×*N*^→*ℝ* measures the training loss made by the mapping function î during the optimization of the training data. In particular, for the supervised machine learning problems based on the neural network, the mapping function can be further formulated in the form

(11) $$\xi_{w}=\left(\sigma_{L}\circ W_{L}\right)\circ \ldots ..\circ \left(\sigma_{1}\circ W_{1}\right),$$

Where *σ*_*l*_ is the activation function, *W* := (*W*_1_…..*W*_*L*_) is the weighting vectors, and *L* donates the number of processing layers in the neural network. As shown in Eq. (11), in the neural network, one of the most important parameters is weights _*W*_, which composites from the weight vector’s entities. Typically, these weighting vectors update during the training process for optimal image reconstruction. Multiple methods have been developed onboard to optimize weighting vectors. Considering that, stochastic gradient descent (SGD) is used to perform this task and diminish the training loss.

**FIGURE 3:**
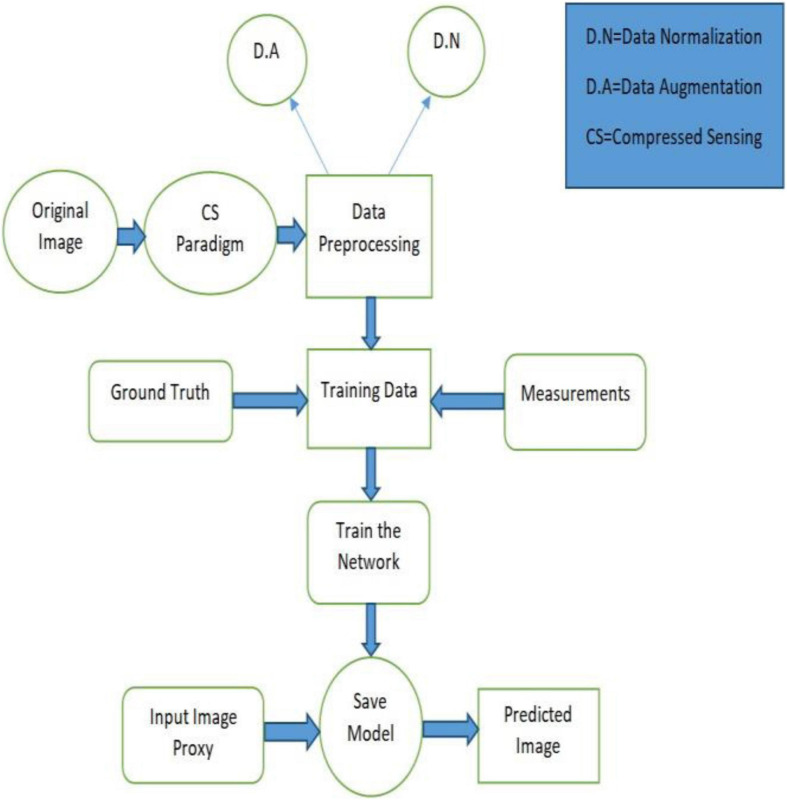
Flowchart of the Deep-PAT method.

In this paper, multiple CNNs, including a simple 3-layer CNN, U-Net, and ResU-Net, are employed. U-Net was initially designed for biomedical image segmentation and used for low-dose CT images (Ronneberger et al., 2015). This network analyzes and processes the training images based on every pixel, hence showing the incredible performance for limited medical datasets. Note that the U-Net’s final layer that was initially used for segmentation needs to be changed for the image reconstruction task. In the case of ResNet and to further improve the performance of the U-Net architecture, a series of residual blocks are stacked together that benefits in term of degradation problems with the help of skip connections within the residual unit and helps to propagate the low- and high-level information of the network. When applied to PAT imaging, CNNs output the artifact-free image using very few measurements. Besides this, the computational cost can also be significantly reduced as compared with traditional iterative algorithms. The proposed method is formulated as follows:

(1) First, CS is applied to high-resolution photoacous ticmouse data (which usually do not have sparse representation) in *ℝ*^*N*^ to generate the measurement vector in *ℝ*^*M*^ (_*M<<N*_) without following the CS prerequisite conditions, i.e., sparsity and incoherence. After the generation of the measurement vector, the image proxy $\hat{V}$ is formulated using the fully connected layer having undersampled artifacts, which eventually weakens the object.
(2) Second, the U-Net–based deep neural network is applied to the image proxy $\hat{V}$ to remove the artifacts and recover the lost information that disappears during the first step. Figure 3 elaborates on the Deep-PAT methodology by flowchart.

### Quantitative Analysis

In this work, the imaging performance of Deep-PAT is quantitatively evaluated by two indicators, i.e., the structure similarity index (SSIM) and peak signal-to-noise ratio (PSNR). The SSIM is a perceptual metric that quantifies image quality degradation and gives a normalized mean value of structural similarity between the two images. The term “structural information” emphasizes the strongly interdependent or spatially closed pixels. These strongly interdependent pixels refer to more important information about the visual objects in the image domain. According to Sara et al. (2019), SSIM can be expressed through these three terms as

(12) $$S\,S\,I\,M\,\left(x,y\right)={\left[l\,\left(x,y\right)\right]}^{a}.{\left[c\,\left(x,y\right)\right]}^{b}.{\left[s\,\left(x,y\right)\right]}^{\gamma }.$$

The above-defined parameters are dependent on three different factors, where *l* characterizes the luminance, which is used to compare the brightness of the predicted and original images; *c* represents the contrast; and *s* is used to compare the structure of both images. Apart from these *a*, *b*, and γ are the positive constants, and *x* and *y* are the original and reconstructed images, respectively. Moreover, the luminance, contrast, and structure are further dependent on the following factors:

(13) $$l\,\left(x,y\right)=\frac{2\,\mu_{x}\,\mu_{y}+c_{1}}{\mu_{x}^{2}+\mu_{y}^{2}+c_{1}},$$

(14) $$c\,\left(x,y\right)=\frac{2\,\sigma_{x}\,\sigma_{y}+c_{2}}{\sigma_{x}^{2}+\sigma_{y}^{2}+c_{2}},$$

(15) $$s\,\left(x,y\right)=\frac{\sigma_{x\,y}+c_{3}}{\sigma_{x}^{2}\,\sigma_{y}^{2}+c_{3}},$$

where *μ*_*x*_ and *μ*_*y*_ are the local means, *σ*_*x*_ and *σ*_*y*_ represent the standard deviation, and *σ*_*xy*_ is cross-covariance for the reconstructed and original images subsequently. Besides this, another indicator (PSNR) is also calculated to validate the image quality as follows:

(16) $$P\,S\,N\,R=10\,\log_{10}\left(\frac{\max_{i\,m\,a\,g\,e}^{2}}{M\,S\,E}\right).$$

## Experimental Materials

This section provides insights into the Deep-PAT method and numerical realization of data processing under the variance conditions.

### Data Set

The available online dataset generated by a full view tomographic scanner having the capability to attain the high-resolution images of a living mouse’s whole body, including the brain, is used (Github, 0000). The scanner comprises 512 individual scanner elements on an 80-mm-diameter ring detection array, which operates on a 5-MHz central frequency, >80% detection bandwidth, 0.37-mm width, and 15-mm height along the elevation direction. PAT is different from CT and MRI imaging modalities and contains optical illumination as well. The photoacoustic signal is excited with a short pulse laser (<10 ns) with a repetition of 15 Hz and 1,680 nm wavelength. Furthermore, after recording the signal from all 512 scanners, the data is simultaneously digitized at 40 megapixels per second. Finally, the data is transferred to a PC *via* ethernet cable to reconstruct using the various methods.

### Data Preprocessing and Network Training

There is a constraint of a large number of data availability in medical applications when using the DL-based method. To overcome the limitation, in this work, data augmentation is used to train the network, which can learn the robustness properties by performing different operations to avoid the overfitting problem. Briefly, before network training, some of the operations are performed, including rotating, which rotates the images to a certain degree; flipping to flip the orientation of the images; and cropping to focus on the features of a certain area of the object. Besides this, to perform smooth learning and converge the network rapidly, a data normalization technique is applied to normalize the dataset between (−1, 1). The dataset comprises 1,130 images after augmentation, which are further split into 80% training and 20% test sets.

The specially designed modality takes the input image *ℝ*^*N*^. It creates the measurement vector *v* (MV), having several measurements *ℝ*^*M*^ using a randomly selected measurement matrix (without fulfilling the RIP property). Then, to boost the dimensionality of the measurement vector and create the image proxy $\hat{V}$ for further processing, a fully connected layer is employed, which is accomplished by multiplying the transpose of a manually designed parameter *K*^*T*^, i.e., $\hat{V}$ = *K*^*T*^*v*. For training, the dataset is converted into pairs of high-resolution images *x*_*i*_ and their corresponding measurements *v*_*i*_. The training dataset *D*_*train*_ = {*x*_1_,*v*_1_}, {*x*_2_,*v*_2_},…,{*x*_*l*_,*v*_*l*_} is fed to U-Net to learn and extract the features of the images, which mainly learns nonlinear mapping from the image proxy to the reconstructed image while the testing dataset *D*_*test*_ = {*x*_1_,*v*_1_},{*x*_2_,*v*_2_},…,{*x*_*s*_,*v*_*s*_} contains the *s* pair of images and their corresponding measurement to test the network learning.

The U-Net in our method has two main portions as shown in Figure 2. The first portion is the encoder part, contributing to the analysis and capturing of the feature data. In contrast, the dimensionally symmetric decoder part serves as the second portion, responsible for accurate localization to acquire the final results based on extracted features from the encoder portion. As in the CNN context, both portions are composed of convolutional layers (CONV). After each of the two convolutional layers, the max-pooling (MP) operation is performed in the encoder part, which halves the image size. Hence, when the data disseminates through the encoder part, the resolution of the data stating deteriorates. On the other hand, the image contraction is reversed due to the usage of the up-sampling layer (UP), which subsidizes the restoration of the image size as in the encoder part. This process is repeated until the output in the decoder part reports the same dimension as in the first layer (encoder part). Moreover, the concatenation layer (CONC) is used to increase the data’s spatial resolution due to multiple down-sampling operations. The Deep-PAT structure with simple U-NET is shown as.

In the U-Net, the convolutional layer kernel size is 3×3 with the stride of 1, the MP layer kernel size is 2×2, and the deconvolution layer kernel size is 2×2 with the stride of 2 used.

**FIGURE 4:**
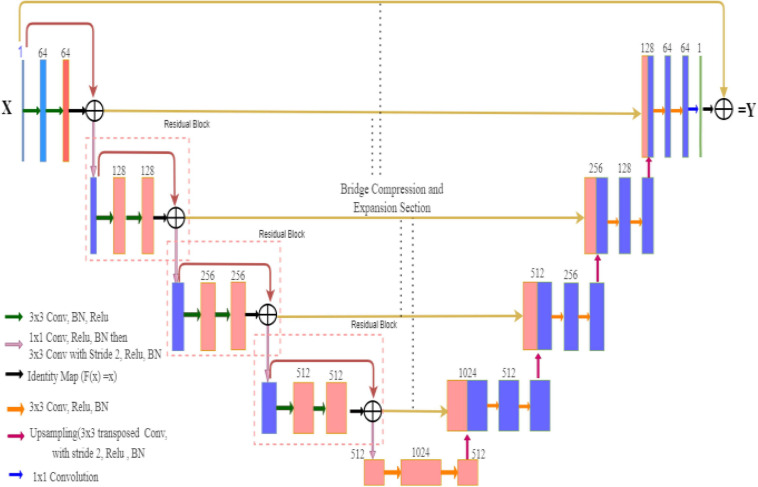
Deep-PAT based on Residual U-Net for image reconstruction.

In the case of ResNet, the encoder block captures the better feature maps in a fine-to-coarse manner and up-samples these feature maps with shortcut connections of residual blocks. The Residual U-Net architecture converts every two convolutional layers at same stage of the U-Net with residual block, whereas 1×1 convolutional operation is needed to match the input and output feature channels in the residual block of the network. In general cases, the depth of the convolutional neural network gradually deepens, the network becomes more and more difficult to train, and the problem of network performance degradation occurs. ResNet further deepens the network by introducing a jump connection structure, solves the problem of gradient disappearance, and improves network performance. The Deep-PAT with ResU-Net is shown in Figure 4.

**FIGURE 5:**
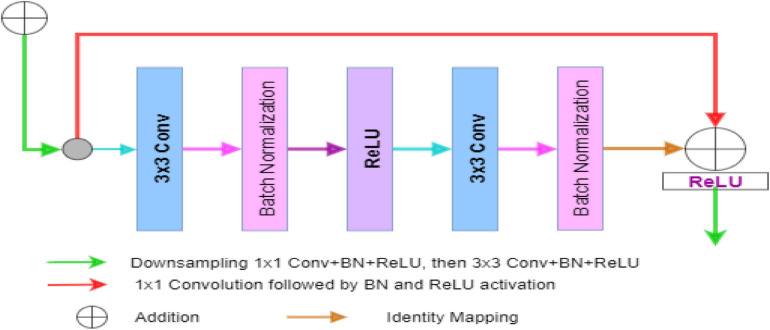
The block diagram represents the encoder block. Each block consists of two_3×3_ convolutional blocks followed by BN and ReLU activation function. Identity mapping is applied to connect the input and output of the encoder block.

Figure 5 is the residual unit, which consists of an identical connection path and a residual path. The residual path is composed of two 3 × 3 convolutional layers and batch normalization (BN) and ReLU (rectified linear units) activation functions, and finally, the results of the two paths are added together to get the output. At the same time, the jump connection does not introduce additional parameters and computational complexity. All the other network parameters are the same as the original U-Net.

Afterward, the networks calculate the loss of training in each iteration or epoch using the ℓ_1_-norm function. To minimize the ℓ_1_ loss function, the ADAM optimizer is used. After each iteration, the weighting vectors are adjusted by back-propagating the loss concerning the parameters, using the stochastic gradient descent method. Here, the learning rate is kept equal to 0.005, and the batch size is equal to 1. The network is implemented on Python with the PyTorch package running on GPU NVIDIA Tesla V100 with CUDA and took 20s for each epoch.

## Results and Discussion

The above-formulated method is applied to three different neural networks (a simple 3-layer CNN, U-Net, and Res-U-Net network). Also, the TV minimization method for sparse data is compared further to demonstrate the imaging performance of the proposed method.

**FIGURE 6:**
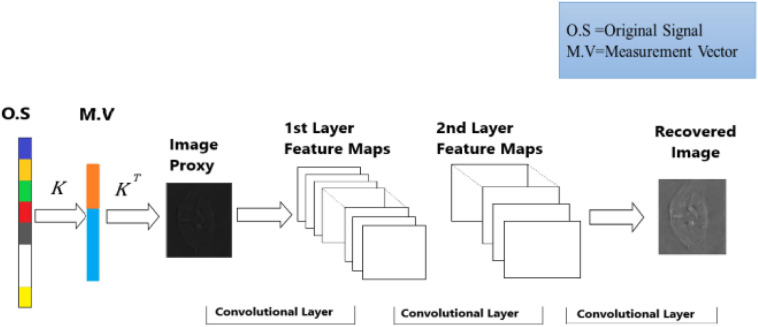
Schematic diagram of 3-layer CNN model.

Briefly, the dataset is divided into pairs (ground truth and measurement matrix), and the CS paradigm is applied to generate a training dataset. Afterward, the output is applied to a simple CNN model having three fully connected convolutional layers. At once, the training is employed and CNN is tested on several unseen images to evaluate the network performance qualitatively and quantitatively under different measurement conditions, i.e., M/N. The 3-layer CNN model is shown in Figure 6.

**FIGURE 7:**
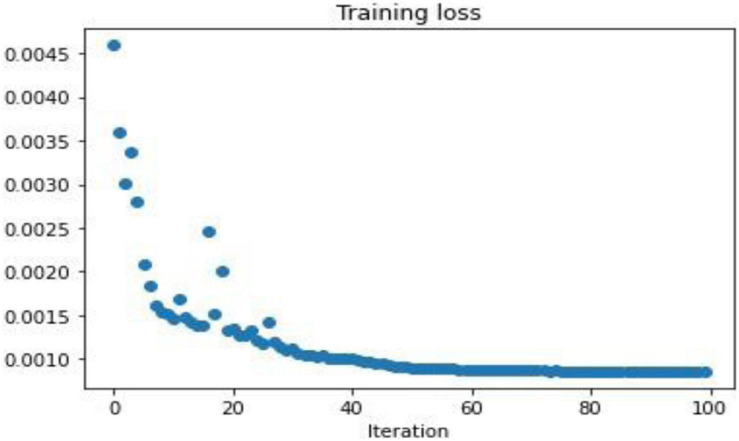
Mean square loss for a simple 3-layer CNN model.

**FIGURE 8:**
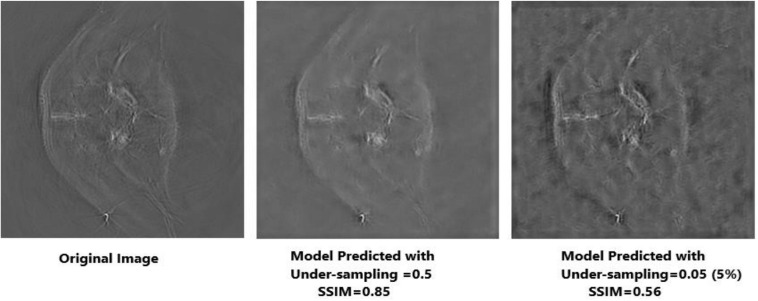
Comparison of different undersampling measurements using 3-layer CNN.

To demonstrate the performance obtained by a simple 3-layer CNN, the comparison is illustrated through qualitative and multiple quantitative means. Figure 7 shows the training loss. Here, the training loss for the simple CNN is calculated by mean square error, and Figure 8 compares the reconstruction results of the simple CNN when using the data with different sampling ratios.

**FIGURE 9:**
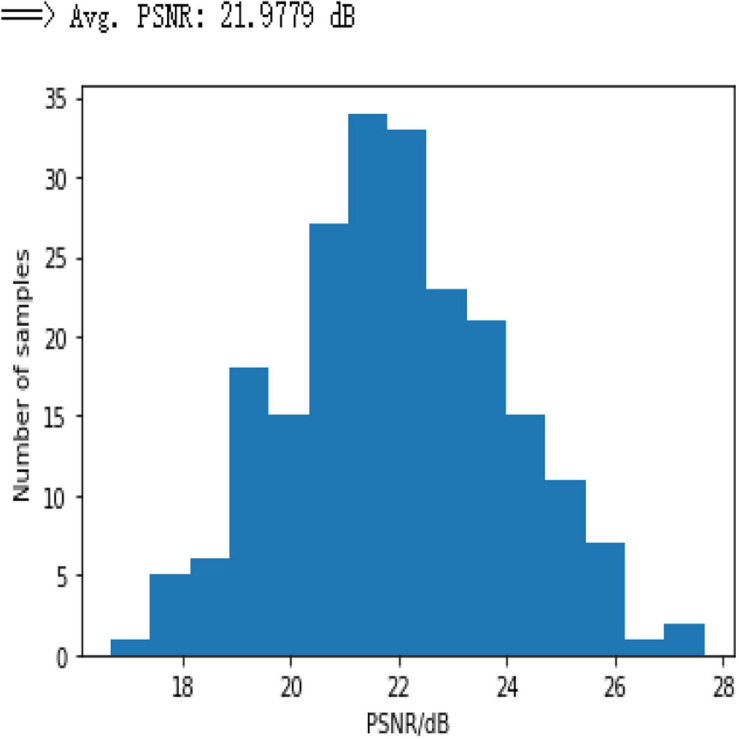
Histogram of PSNR for undersampling ratio = 0.3 on simple CNN network.

Based on the results in Figure 8, it can be seen that the network performance is not satisfactory due to the usage of a limited amount of data and few weights as the CNN network does not work well for limited information, and the efficacy of the simple CNN to extract the feature maps is insufficient to reconstruct the efficient results. Moreover, Figure 9 shows the PSNR of the test dataset for 3-layer CNN with average PSNR.

**FIGURE 10:**
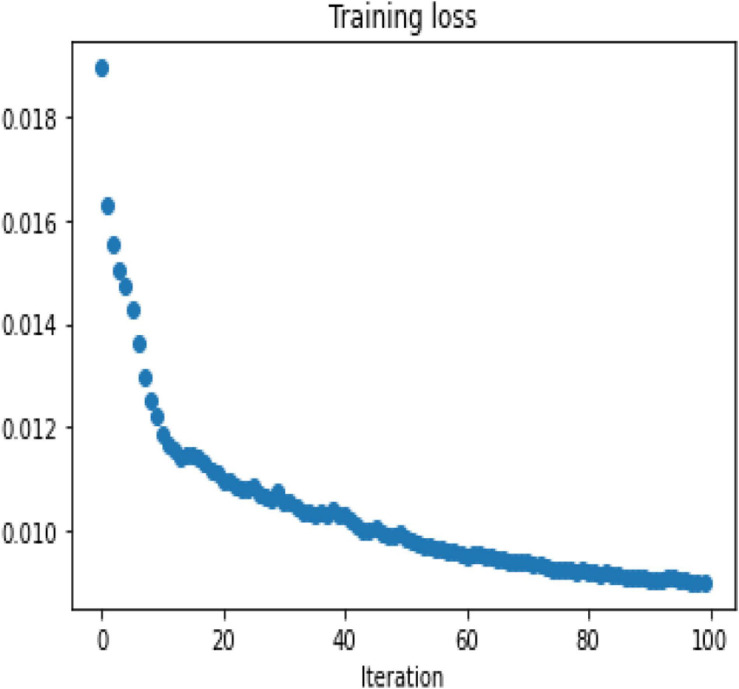
Training loss for Deep-PAT (U-Net) network.

Afterward, the same procedure is performed using the specially designed Deep-PAT with the U-Net and Residual U-Net networks, as explained in the methodology section, to reconstruct the images under different sampling conditions. The same abovementioned random data is converted into the test- train sets that further comprise measurements and original images. The mean square training loss for U-Net is shown in Figure 10.

**FIGURE 11:**
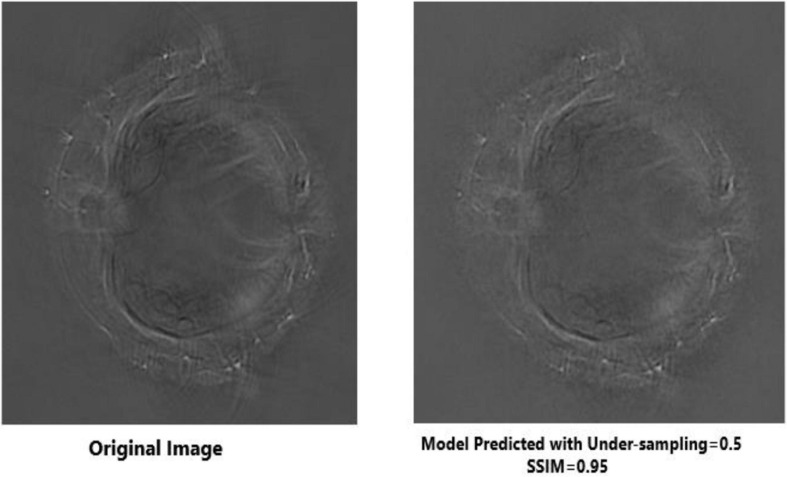
Visual comparison between the original image (left) and reconstructed image (right) using half of the original measurement *via* Deep-PAT (U-Net).

**FIGURE 12:**
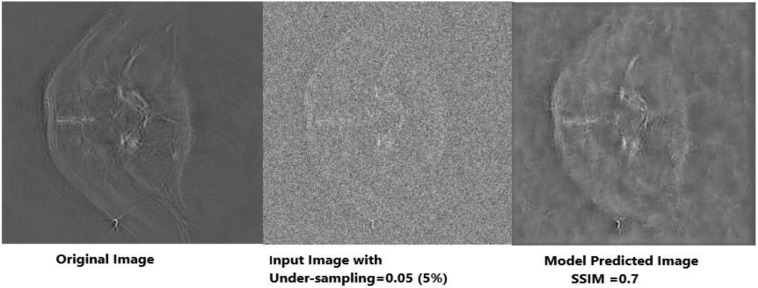
Visual representation of image reconstruction using Deep-PAT (U-Net). The leftmost image represents the original image; the middle image is the input image with a disturbing object, and the rightmost shows the predicted image with considerable SSIM.

The outcomes generated by Deep-PAT (U-NET) are shown in Figures 11, 12.

**FIGURE 13:**
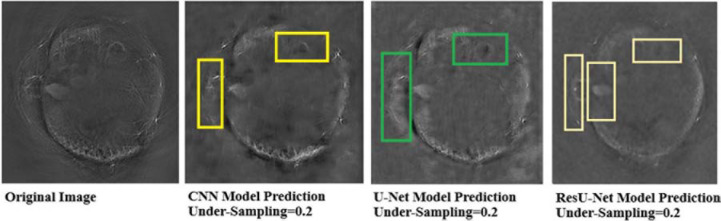
Comparison of two methods (the U-Net and the simple 3-layer network) based on edges and luminance recovery. The left image represents the original image; the middle image is recovered using Deep-PAT (U-Net), and the right image shows the visual representation of CNN prediction.

**FIGURE 14:**
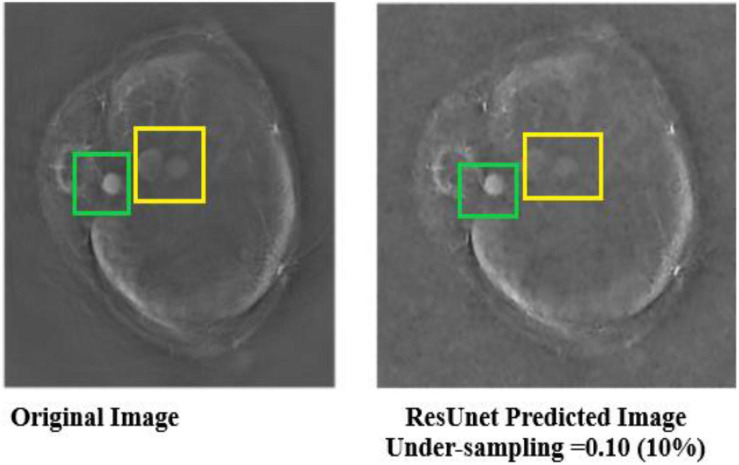
Predicted results of Res-U-Net in the worst scenario. The left images are labeled as the original image, and the right image shows the visual representation of Res-U-Net output.

As mentioned, the main concern is to reconstruct the photoacoustic images by using very low measurements, which is a really complicated and challenging task in the presence of the disturbing object. Deep-PAT is set to perform this task with efficiency as shown in Figures 11–14.

It can be seen that the Deep-PAT (U-Net) is capable enough to reconstruct the photoacoustic data with high efficiency based on edges, luminance, and contrast. The enlarged view represented by the green box visualizes that the proposed network not only removes the undersampled artifacts, but also recovers the missing information and edges of the randomly selected image (Figure 13). Besides this, the performance of Res-U-Net outperformed the other two networks with a slight difference in qualitative and quantitative analysis as compared with simple U-Net. The worst scenario of Res-U-Net is still able to recover the organs of the body and remove most of the over-smoothness in the predicted image that appears in U-Net and simple CNN as shown in Figure 14.

It can be noticed from the results that some over-smoothing is presented that causies some details to be removed. This is due to the short length of features in the direction of undersampling, which are difficult to identify due to the worst nature of the input image. Overall, the experiments show that Res-U-Net outperforms the U-Net and simple 3-layer CNN network as seen in Figure 14. This leads to the comparison with different neural networks without following CS prerequisite conditions and helps to choose the best one. The next section of the paper presents a comparison with the sparsity-based method.

**FIGURE 15:**
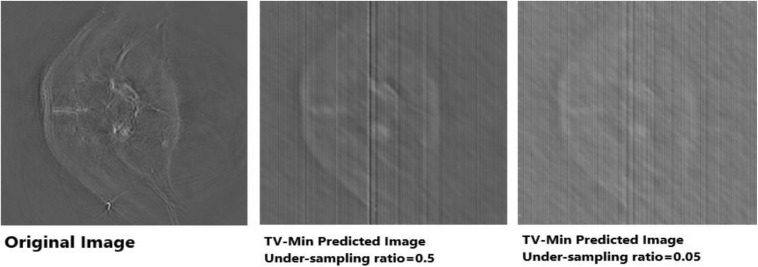
Reconstruction results obtained by TV-minimization with different undersampling ratio. The leftmost is an original image; the middle image is recovered by the TV-Min method with an undersampling ratio = 0.5, and the rightmost image shows the output with undersampling ratio = 0.05 (5%).

Apart from neural networks, to support the Deep-PAT method, the sparsity-based TV minimization is also implemented. Referring to Figure 15, it can be seen that Deep-PAT (all networks) outperforms the sparsity-based TV minimization algorithm for this kind of complex dataset. The main reason may be that the TV-based model is well suited to the recovery of only a few types of images (i.e., with piecewise constant) (Dobson and Santosa, 1994; Chambolle and Lions, 1997; Cand‘es et al., 2006). Later, many authors proposed the solution by minimizing the gradient’s nonconvex function, which increases the image quality. However, the instability is always here for this kind of method.

**FIGURE 16:**
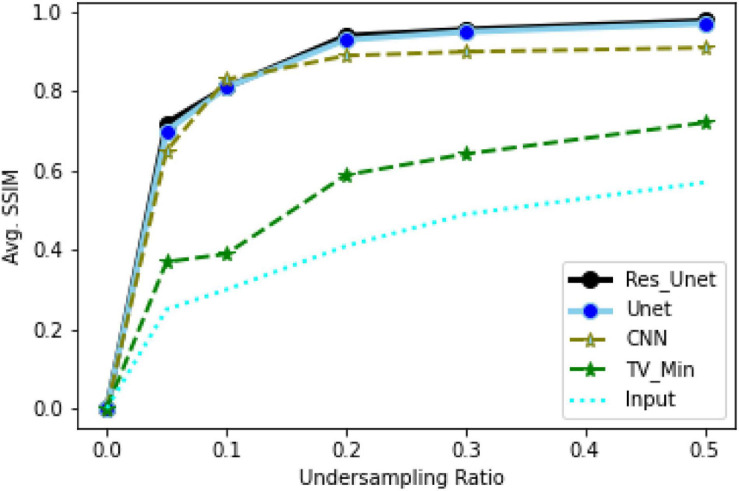
Average SSIM comparison between input, CNN, Deep-PAT, and TV minimization.

**FIGURE 17:**
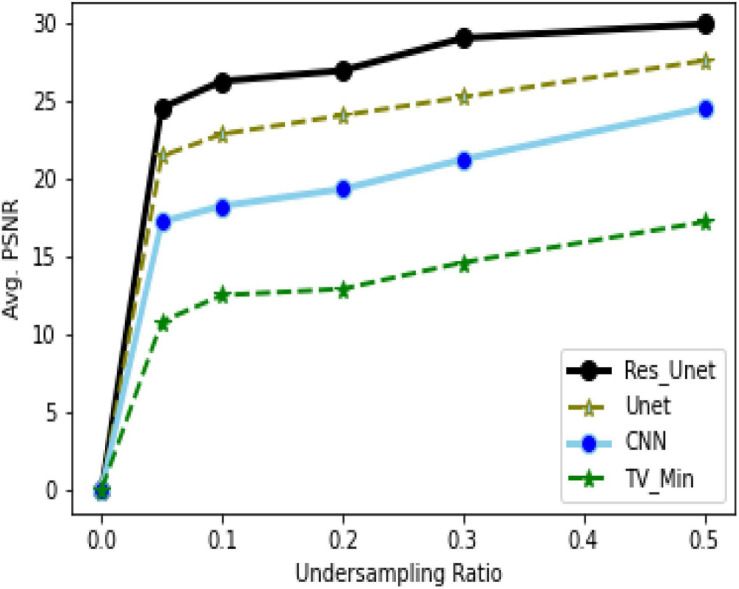
Average PSNR (dB) for different sampling rates and networks.

To quantitatively evaluate the imaging performance obtained by different methods combining with the different undersampling ratio data, the SSIM and PSNR are calculated, and the corresponding results are shown in Figures 16, 17 and Table 1. The results further validate the performance of the proposed method.

**TABLE 1:** Statistical analysis of both methods regarding evaluation parameters.

Undersampling ratio	Average CNNPSNR (dB)	Average + SD CNN SSIM	Average + SD U-Net PSNR (dB)	Average + SD U-Net SSIM	Average + SD Res-U-Net PSNR (dB)	Average + SD Res-U-Net SSIM	Average + SD Input SSIM	Average TV-minimization SSIM	Average TV-minimization PSNR (dB)
0.5	24.5 ± 0.142	0.91 ± 0.005	27.53 ± 0.097	0.97 ± 0.006	29.88 ± 0.089	0.97 ± 0.007	0.57 ± 0.376	0.72 ± 0.091	16.48 ± 0.201
0.3	21.9 ± 0.201	0.90 ± 0.005	25.20 ± 0.101	0.95 ± 0.05	28.98 ± 0.081	0.95 ± 0.005	0.49 ± 0.5154	0.67 ± 0.132	14.78 ± 0.295
0.1	18.22 ± 0.150	0.83 ± 0.030	22.83 ± 0.204	0.81 ± 0.070	27.22 ± 0.106	0.80 ± 0.090	0.30+0.485	0.41 ± 0.514	12.91 ± 0.352
0.05	17.21 ± 0.149	0.65 ± 0.021	21.41 ± 0.186	0.70 ± 0.045	24.51 ± 0.176	0.76 ± 0.098	0.25+0.6452	0.38 ± 0.317	11.93 ± 0.391

*PSNR, peak signal-to-noise ratio; SSIM, structural similarity index; RT, reconstruction time; SD, standard deviation.*

Based on the average^1^ SSIM and PSNR visualized in Figures 16, 17, it can be seen that how bad the input is, having very low SSIM, whereas the TV minimization (sparsity-based method) shows abysmal performance with just SSIM = 0.72 for the 50% undersampling case. In contrast, the simple 3-layer improves the performance but not at a satisfactory level due to biased recovery and over-smoothing the image, having SSIM = 0.65 for the 5% undersampling case. Meanwhile, simple U-Net performs better as compared with the previously discussed methods with SSIM = 0.70 for the 5% undersampling scenario but experiences the over-smoothing problem as well. Besides this, Res-U-Net outperformed all three methods in terms of qualitative and quantitative analysis and removes the over-smoothing problem to a great extent even in the worst-case scenario having SSIM = 0.76 for the 5% undersampling case.

## Conclusion

In this paper, the specially designed Deep-PAT is proposed for the reconstruction of experimental photoacoustic whole body mouse data without taking the prerequisite conditions (sparsity and incoherence) of CS into consideration. The dataset is created by scanning the whole body, including the brain of the mouse. The proposed method breaks the bottleneck in using the CS domain for recovery or reconstruction of photoacoustic medical images.

The methodology is implemented on photoacousticmouse data to validate the theoretical concerns. This approach iscompared with a classical method (TV minimization), which strictly obeys the CS-based sparsity and RIP conditions.

For future work, the more advanced networks could be designed to reconstruct a brain-wide vascular network for neural imaging to get more detailed information with a low processing cost. Additionally, in the methodology context, U-Net could be computationally more efficient using a skip connection, which would only process the essential features and discard the unnecessary data from the images (diminishing the sparsity conditions) (Drozdzal et al., 2016; Yamanaka et al., 2017). Moreover, a fundamental improvement will be to refine and apply new model architectures, such as generative adversarial networks (Hussein et al., 2020), which may yield modest performance gains. Apart from this, the tailored deep-learning models specifically for PAT or PAM for neural imaging could be designed with the amalgamation of CS.

## Data Availability Statement

Publicly available datasets were analyzed in this study. This data can be found here: https://github.com/ndavoudi/sparse_artefact_unet/tree/master/dataset.

## Author Contributions

HS developed the theoretical formalism and performed the analytic calculations. HS and AK performed the numerical simulations. XL and MI contributed to the final version of the manuscript. DT supervised the project. All authors contributed to the article and approved the submitted version.

## Conflict of Interest

The authors declare that the research was conducted in the absence of any commercial or financial relationships that could be construed as a potential conflict of interest.

## Footnotes

1 The network gives the different SSIMs (in a decimal range) for every image in the test set based on the structure of the image. Hence, in order to normalize the results, the average command takes the entire test set’s PSNR and SSIM and gives one average value for the whole set at a particular undersampling ratio.
