# Supplementary material for: A Deep Learning Approach for the Photoacoustic Tomography Recovery From Undersampled Measurements
Source: Front Neurosci. 2021 Feb 24;15:598693. doi: 10.3389/fnins.2021.598693 (PMC7943731; doi:10.3389/fnins.2021.598693)
Supplement: Supplementary file 1 [file Data_Sheet_1.pdf]

SUPPLEMENTARY FIGURES

Dataset Acquisition:

The schematic diagram of the experiments and data-acquisition [26]

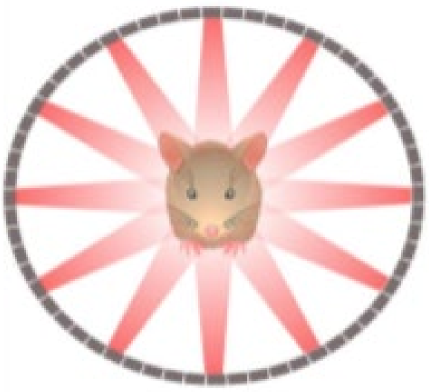

Supplemental Fig.1. The cross-sectional (circumferential) light illumination configuration with 12-arm fiber bundle on whole mice body

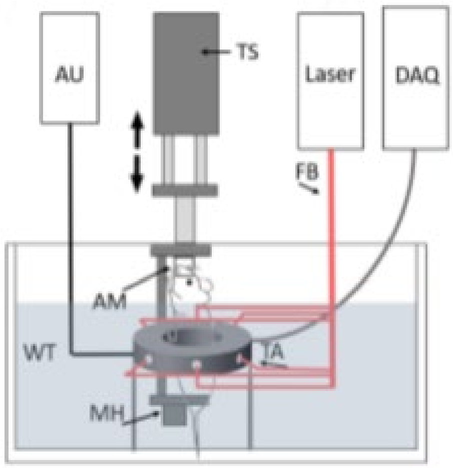

Supplemental Fig.2 Lay-out of the complete full-ring array imaging setup. AU – Anesthesia unit, TS – Translation stage, DAQ- Data acquisition, FB – Fiber bundle, AM- Anesthesia mask, WT – Water tank, MH – Mouse holder, TA – Transducer array
